# On lightmyography based muscle-machine interfaces for the efficient decoding of human gestures and forces

**DOI:** 10.1038/s41598-022-25982-w

**Published:** 2023-01-06

**Authors:** Mojtaba Shahmohammadi, Bonnie Guan, Ricardo V. Godoy, Anany Dwivedi, Poul Nielsen, Minas Liarokapis

**Affiliations:** 1 New Dexterity Research Group, Department of Mechanical and Mechatronics Engineering, University of Auckland, Auckland, 1010 New Zealand; 2 Chair of Autonomous Systems and Mechatronics, Department of Electrical Engineering, Friedrich-Alexander-Universität Erlangen-Nürnberg, 91052 Erlangen, Germany; 3 Auckland Bioengineering Institute, The University of Auckland, Auckland, 1010 New Zealand

**Keywords:** Biomedical engineering, Mechanical engineering

## Abstract

Conventional muscle-machine interfaces like Electromyography (EMG), have significant drawbacks, such as crosstalk, a non-linear relationship between the signal and the corresponding motion, and increased signal processing requirements. In this work, we introduce a new muscle-machine interfacing technique called lightmyography (LMG), that can be used to efficiently decode human hand gestures, motion, and forces from the detected contractions of the human muscles. LMG utilizes light propagation through elastic media and human tissue, measuring changes in light luminosity to detect muscle movement. Similar to forcemyography, LMG infers muscular contractions through tissue deformation and skin displacements. In this study, we look at how different characteristics of the light source and silicone medium affect the performance of LMG and we compare LMG and EMG based gesture decoding using various machine learning techniques. To do that, we design an armband equipped with five LMG modules, and we use it to collect the required LMG data. Three different machine learning methods are employed: Random Forests, Convolutional Neural Networks, and Temporal Multi-Channel Vision Transformers. The system has also been efficiently used in decoding the forces exerted during power grasping. The results demonstrate that LMG outperforms EMG for most methods and subjects.

## Introduction

Rapid breakthroughs in robotics have highlighted the importance of effective interaction and communication between humans and machines, with various robotic devices being introduced to a range of industries such as housing, hospitality, and medical devices^1–3^. Traditionally, a user makes a decision and communicates it to the device via an interface, and the device responds only to the provided command. More advanced systems, on the other hand, only need raw data from the user to make decisions automatically using machine learning (ML) techniques ^4^. This makes the human-machine interface (HMI) a crucial part of the system both for data acquisition and communication.

Various HMIs have been developed employing various methods and concepts based on the specific needs of the user^5–7^. Handheld controllers are the most prevalent type of HMI, and they are widely used in various applications due to their ease of learning and operation^8,9^. However, they occupy the user’s hands, can induce fatigue with long-term use, and may not be practical and intuitive enough for devices such as prostheses. Another method of controlling a robotic device is by using vision-based systems^10,11^. Such systems are relatively reliable and typically do not interfere with the user’s workspace; nonetheless, they are susceptible to occlusion and changes in environment lighting. Human-machine interaction is also possible through voice commands^12,13^. This is a popular interface, although, as with vision-based systems, noise from the surroundings can interfere with communication. Wearable devices such as eyeglasses, gloves, and electroencephalography (EEG) helmets are other types of HMIs that can control a machine based on the user’s movements or biological signals^14–17^. They also exist in the form of armbands that capture data from the user’s forearm and use machine learning techniques to predict the intended gestures of the user. Recent research has applied Deep Learning approaches to analyze several biological signals. These methods rely on Deep Learning’s ability to extract high-level features and learn hierarchical representations from many low-level input samples^18^, resulting in an increasingly complex and robust system^19^.

Surface electromyography (sEMG) can be used in wearable devices to measure the electrical activity of the user’s muscles^20^. The sEMG sensors are able to detect tiny amounts of generated electricity in a target muscle group during the execution of a task. EMG-based interfaces generally need complex electronics for data acquisition and processing, the use of gel-based electrodes, appropriate muscle group selection, and precise placement of the EMG sensors, making their integration into portable devices complicated^21^. Alternatively, human-machine interfacing armbands can use forcemyography (FMG) to capture the skin displacement generated by the muscle contraction and predict the corresponding hand gestures^22–25^. Compared to EMG-based devices, FMG is less prone to the confounding effects of sweating and moisture. The primary disadvantage of FMG is that it does not detect muscular activation directly; relying instead on muscle volume changes during contraction and relaxation, which results in a force difference between the sensor and the muscle^26^.

**Figure 1:**
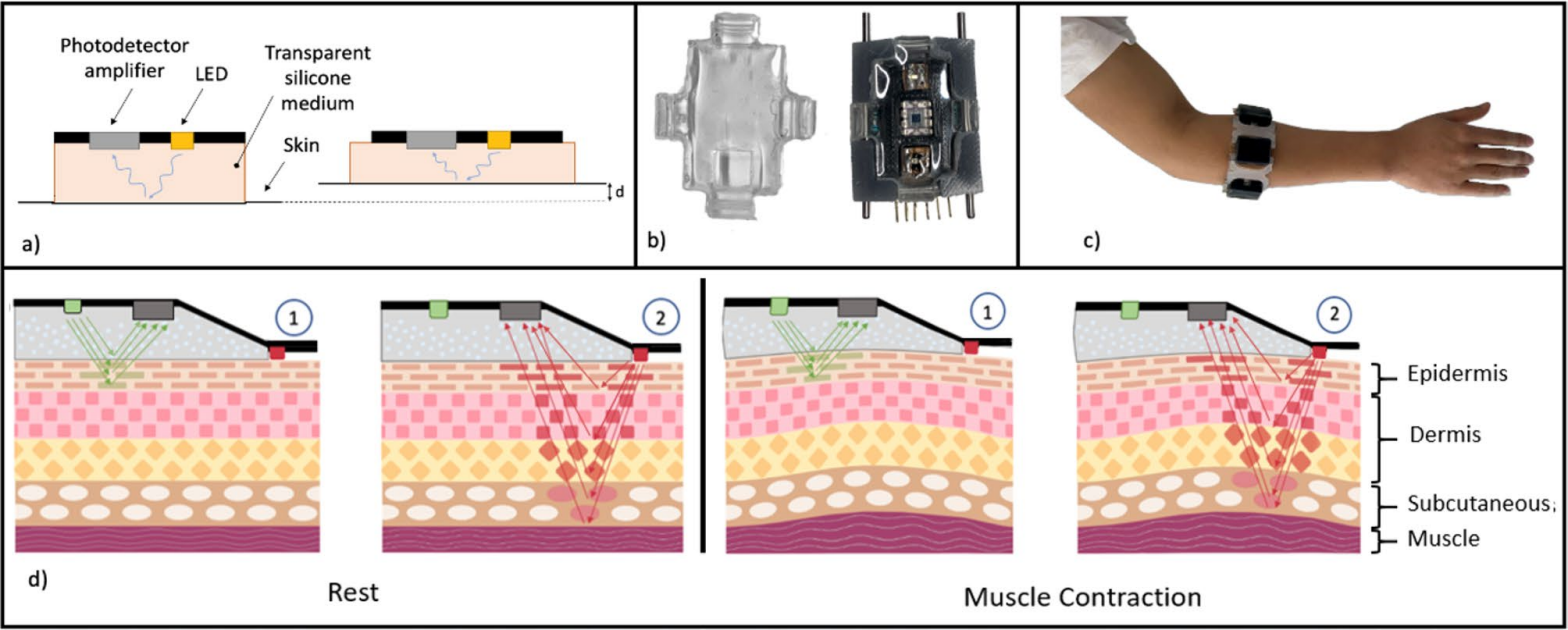
Working principle of the Lightmyography. (**a**) shows a simple module with one LED and one photodiode. (**b**) shows the developed modules of the proposed armband that are equipped with two photodiodes and a single photodetector. (**c**) shows the proposed armband worn on the upper half of a human forearm. Finally, (**d**) shows the working principle of the Lightmyography method, where different lights of different wavelengths penetrate the skin in different depths and get reflected on the tissue before they travel back to the skin surface to be captured by the photodetector. Muscle contractions become tissue deformations that affect the light luminosity that gets captured by the photodetector, and thus can be easily detected.

In this research paper, we investigate and propose a new human-machine interfacing technique called Lightmyography (LMG) and we assess its applicability in capturing the muscle activity of the human forearm muscles. An LMG interface employs light emitting diodes (LEDs) that release light through a silicone medium towards the skin, and the reflected light is measured using appropriate photodetectors that are located next to the LEDs. When the targeted muscle group contracts during a gesture, it compresses or decompresses the surrounding tissue and the silicone medium attached to the skin on top of it. This compression/decompression of the tissue and the silicone medium changes the light luminosity recorded by the photodetectors. Then, appropriate machine learning methods can be used to decode the gestures that the user wants to execute or the forces exerted based on the signals recorded by the different photodetectors.

Moreover, in this paper, we also investigate the effects of light wavelength, reflecting surfaces, silicone color, and silicone stiffness and thickness on the performance of the LMG-based decoding models. The outcomes of these comparisons and analyses are then used for the design and development of a complete LMG armband that can detect various user hand gestures and force exertion patterns. The armband is produced using 3D printing and molding, and consists of five green LEDs, five IR LEDs, and five photodiodes. Various machine learning methods are employed to produce LMG-based models that can successfully decode five distinct gestures, validating the effectiveness of the LMG technique and proposed armband. Additionally, the armband’s ability to perform LMG-based force decoding during clenching was evaluated by training and validating the performance of appropriate regression models.

The rest of this paper is organized as follows: Section II presents the design of the LMG sensing modules, Section III presents the design of the proposed LMG armband, Section IV presents the experimental results and discussions, while Section V concludes the paper.

## Methods and design considerations

In this section, we present the methods and the design considerations necessary for developing an efficient Lightmyography-based gesture and force decoding framework. Each LMG sensor combines emitters, photodetectors, and silicone layers that are used in between the LEDs and the human skin. The effect of each of these components on the sensor’s performance is investigated. Then, based on the results of these experiments, we design an LMG armband to perform gesture prediction and force decoding. Figure 1 illustrates the working principle of LMG, while Table 1 summarizes the LMG sensing module components tested in this section and presents the key findings.

### Experimental investigation of the effect of the LMG design parameters

**Figure 2:**
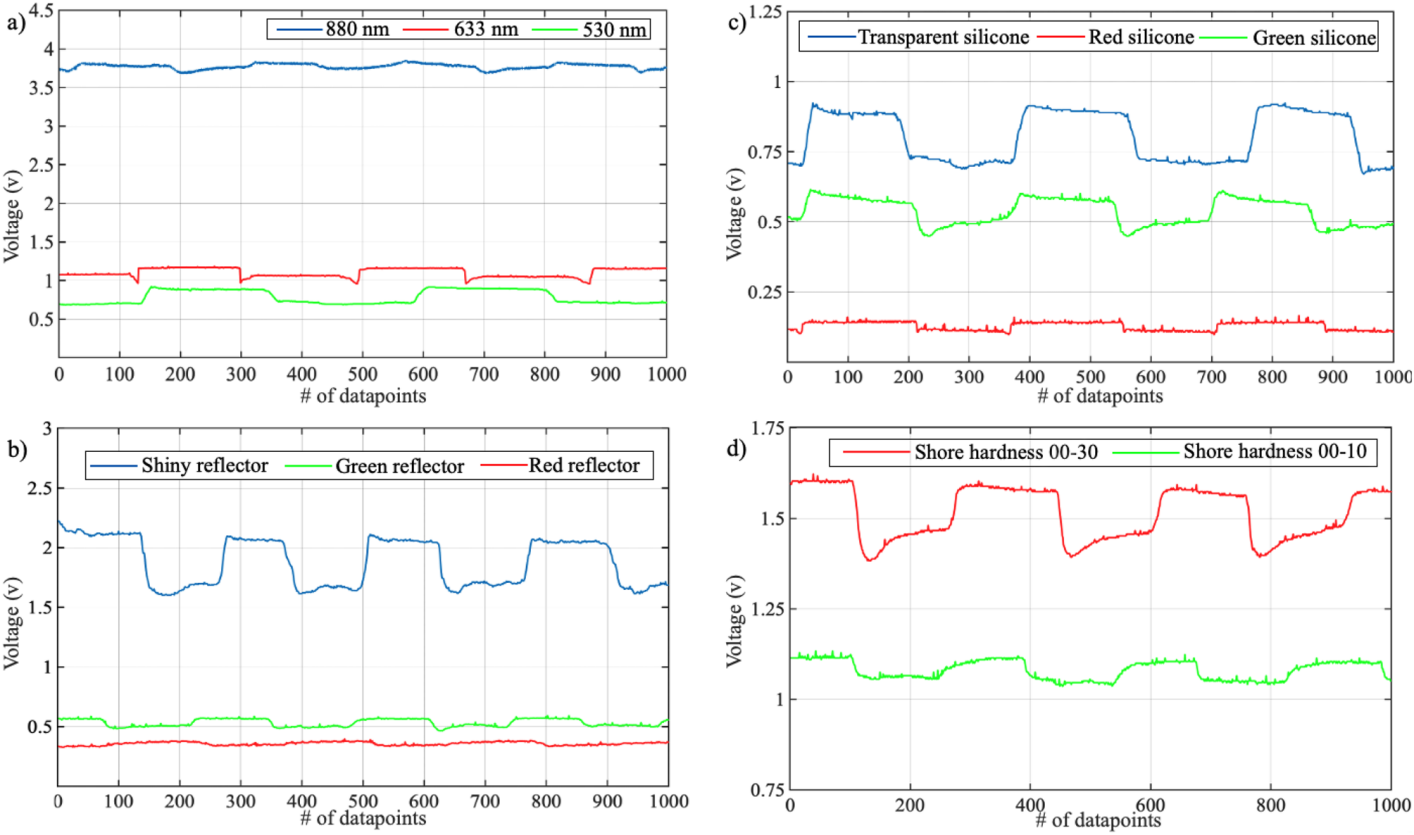
Exemplar graphs of the experiments conducted. (**a**), presents a comparison between the acquired data using three different light wavelengths: 530 nm, 633 nm, and 880 nm. (**b**), presents a comparison between the acquired data using three different flexible reflective surfaces: green, red, and shiny. (**c**), presents a comparison between the acquired data using three different colors for the silicone medium: green, red, and transparent. Finally, (**d**), presents a comparison between the acquired data using two different stiffness levels for the silicone layer.

Many parameters can affect the performance of a single LMG sensing module. We conducted experiments to understand the effect of wavelength, silicone color, silicone thickness, silicone stiffness, and surface reflection. These are the initial, proof-of-concept experiments that we did to conceptualize the idea and understand the working principles. In all of these experiments, one module is placed on the posterior forearm of a human subject at the intersection of the extensor digitorum and extensor carpi ulnaris muscles. The subject then performs a power grasp gesture in pronation configuration. To make sure that the location of the modules is the same between different experiments, a location mark was placed on the forearm of the subject. In addition, to minimize the effect of environmental lighting, all experiments were conducted in a dark room. Figure 2 shows the results of these experiments.

#### Effect of wavelength

The wavelength of the emitted light plays an important role in the performance of the device. Changes in the wavelength can significantly change the way emitted light interacts with the skin, and can significantly affect light absorption and reflection. Optical characteristics of human skin have been investigated in numerous studies^27–30^. Most of these studies suggest that, by increasing the wavelength in the range of 300 nm to 1000 nm, the reflectance coefficient of the human skin increases as well.

**Figure 3:**
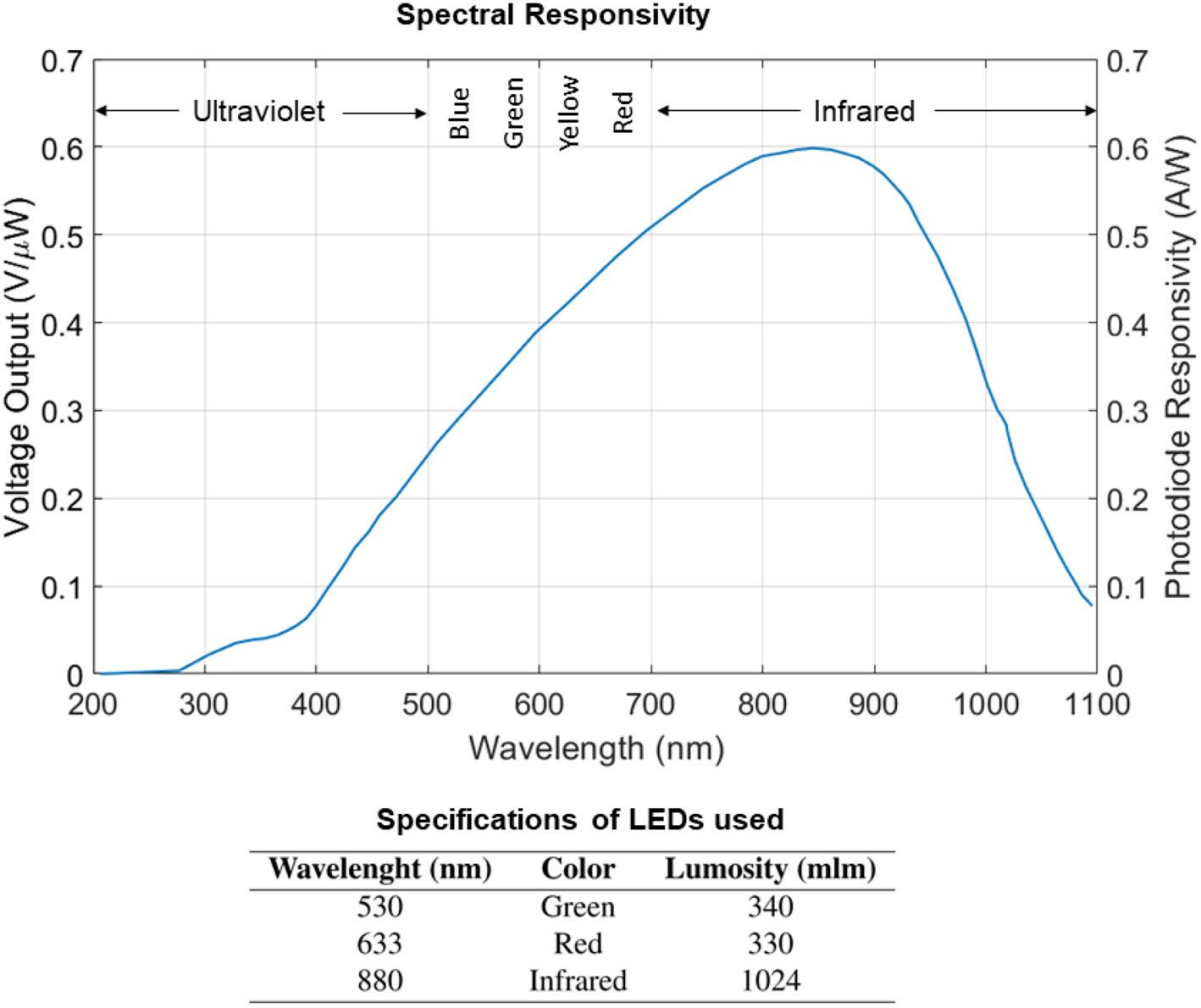
Characteristics of the analyzed LEDs and photodiode^34^.

Although the reflectance changes with different skin pigmentation, in a research by Baranoski et al.^31^ it was shown that the reflectance of both lightly pigmented and darkly pigmented skins change in almost the same manner. To investigate the effect of various wavelengths, we decided to use three different levels: 550 nm, 630 nm, and 850 nm. To detect the reflected light, we use Texas Instruments’ OPT101 Monolithic Photodiode and Amplifier, an all-in-one package with maximum responsivity at 850 nm. These wavelengths were chosen to represent a green (550 nm), a red (630 nm), and an infrared (850 nm) source of light. Figure 3 shows the relationship between the photodiode responsivity and wavelength. It also shows the specifications of the LEDs. Since the sensor responsivity is different at different wavelengths we can’t directly compare the readings in Fig. 2. The same logic applies to the luminosity of the LEDs. To be able to compare the signal values of the experiments, independently from the sensor responsivity and LEDs luminosity, we define the performance index *I*, which is calculated by

1 $$\begin{aligned} I = \frac{\Delta S}{{\Phi _{v}}\cdot R_{p}} , \end{aligned}$$

where $R _{p}$ is the photodiode responsivity at the LED dominant wavelength, $\Phi _{v}$ is the luminosity of the LED, and $\Delta S$ is given by

2 $$\begin{aligned} \Delta S = \left| \bar{S_{g}} - \bar{S_{r}}\right| , \end{aligned}$$

where $\bar{S_{g}}$ is the mean value of the signal during gesture and $\bar{S_{r}}$ is the mean value of the signal during rest. The corresponding *I* values are 0.024, 0.153, and 0.39 for 880 nm, 633 nm, and 550 nm, respectively. It can be concluded from these results that, although the signal magnitudes are higher with red and IR LEDs, the value of the signal during the gesture and at rest, is more distinguishable with the green LED. These results are in agreement with other studies in which authors investigated the effect of wavelength in photoplethysmography devices^32,33^. This is probably due to the lower tissue penetration depth achieved by smaller wavelengths. This suggests with its higher penetration, IR light can give us information additional to what we get with green LEDs (e.g., configuration information of deeper tissue underneath the skin).

#### Discussion of the effect of silicone properties

In this section, we discuss the effect of the different silicone properties based on the results provided in Fig. 2.

*Silicone Color* Initially, we analysed the effect of silicone medium color on the quality of signal. In this experiment we compared a light green silicone, a light red silicone, and a transparent silicone. These colors were chosen to investigate if there was any difference in the signal when the silicone color matches the LED color compared to a different color or a transparent medium. For all of these experiments, we used a green 530 nm LED. The green LED performed better with the green silicone medium compared to the red silicone medium. However, a noticeable improvement in signal to noise ratio is not evident in any of them, and a clear transparent silicone medium provides the most distinguishable signal, as shown in Fig. 2.

*Silicone Stiffness* Compression and decompression of the silicone medium during the execution of a gesture is a function of its stiffness. To investigate this, we created two different modules with silicone Shore hardnesses of 00-10 and 00-30 and tested them using a 530 nm green LED. The stiffer silicone (00-30) shows a better signal response with a performance index (*I*) of 0.272 compared to the softer silicone (00-10) with *I* of 0.161. This is probably because, with softer silicone (00-10), since initially the circuit board is compressed into the silicone medium, the medium reaches its maximum achievable compression before the gesture starts, and no more compression can occur during the gesture. Thus, the softer silicone is not the best option for green LED.

*Silicone Thickness* To investigate the effect of silicone layer thickness, we tested modules with silicone thicknesses of 3 mm, 5 mm, and 7 mm. During the experiments we noticed that, with green LEDs, a certain distance from the skin (5 mm) was necessary to get the best results. It seems that the effect of silicone thickness is dependent on the wavelength of the LED. With the green LED, silicone of 5 mm thickness offered the most easily distinguishable rest and gesture periods in the LMG signal. With the IR LED, the thinnest silicone provided better signals compared to the other two silicones. Best results for the IR LED were obtained when the sensors were placed directly on the human skin.

#### Discussion of the effect of reflective surfaces

To avoid having to cope with the individual-specific variations of human skin characteristics, such as reflectance, roughness, pigmentation, etc., it is possible to use reflective materials embedded in the sensing module. To investigate this, we compared flexible green and red surfaces with moderate reflectiveness, and a shiny aluminum tape with much higher reflectiveness. For this experiment, we used a green 530 nm LED, and transparent silicone material. As expected, the red surface reflected little of the emitted green light and had a very poor performance. With the shiny surface, the value of the signal response was much higher than the other two surfaces, with active and rest regions easily distinguishable. Please see Fig. 2 for more details.

**Table 1:** Summary of each tested LMG sensing module component, showing the best and worst option for each property. A comprehensive description of the findings is also included.

LMG module property	Performance			Findings
	Best	Mid	Worst	
Wavelength (nm)	**530**	633	880	The 530 nm LED showed the highest performance index. Low wavelengths have low tissue penetration depth, reflecting cleanly on the skin surface. Higher penetration depth provides additional information on the muscle contraction and tissue deformation
Silicone colour - green LED	**None**	Green	Red	The most distinguishable signal was noted with transparent silicone, as the medium does not significantly absorb or reflect light
Silicone stiffness (Shore hardness) - green LED	**00-30**	00-10	N/A	The stiffer silicone performed better compared to the softer silicone, as the softer silicone reached its maximum compression before the gesture execution
Silicone thickness - green LED (mm)	**5**	3	7	The 5 mm silicone provided the best thickness for the photodetector as 3 mm is too close for a distinguishable signal and 7 mm is too far for getting adequate luminosity
Silicone thickness - IR LED (mm)	**0**	3	5	Due to the properties of IR LEDs, closer skin proximity resulted in better skin penetration and less environmental lighting noise
Reflective surfaces - green LED	**Shiny**	Green	Red	A shiny reflective surface performed best - as expected - since most light is reflected rather than absorbed

Significant values are in [bold].

### Armband design

Based on the experiments performed in investigating the effect of various design parameters, a complete LMG armband was designed and developed to collect data from different muscle groups of the human upper forearm. The proposed armband consists of five LMG sensing modules. Additional modules may be added or removed, however, five modules were used in this study to comfortably fit around the participants’ forearms, limited by forearm size. In order to collect data from different tissue depths above each muscle group, we employ two LEDs of different wavelengths in each sensing module, one green and one IR, with the photodiode amplifier located at the center of the sensing module. The green LED stays at a 5 mm distance from the skin while the IR directly touches the human skin. We then turn these LEDs on and off consecutively with an interval of 125 ms, while constantly reading the light measured by the photodetector. This allows us to interpret the acquired data that is relevant to each individual LED. The light from the green LED doesn’t penetrate the skin further than the epidermis. On the other hand, the IR light with a longer wavelength can reach all the way to the subcutaneous tissue. The IR light also interacts with other layers such as the dermis when passing through them.

Fig. 1 shows the components of a single sensing module and the LMG working principle. We use a silicone cover to pretension the sensing components so as to ensure that the transparent silicone and IR LED achieve good contact with the human skin. The material for this cover is a silicone with Shore A hardness of 10. We also use silicone pigmentation to dye this part black so as to avoid environmental lighting from reaching the photodiode amplifier, affecting the measured signals. For the transparent silicone, we use a different silicone with Shore A hardness of 15. Elastic bands made out of the same material as the silicone cover are used to connect the modules together and form a complete LMG armband.

**Figure 4:**
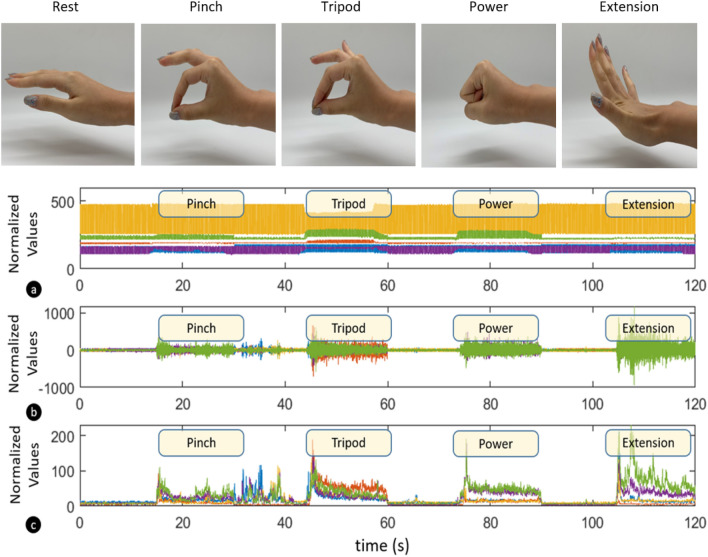
Normalised LMG and EMG measurements during pinch, tripod, power, and extension gesture. (**a**) shows the activation values for the LMG sensors, (**b**) shows the raw EMG activations, while (**c**) shows a feature extracted value (Root Mean Square - RMS value) of the EMG signals.

## Experiments

In order to experimentally validate the performance of the proposed LMG armband, we performed a set of experiments to collect data from ten participants and perform LMG-based gesture classification and force decoding.

### Comparison of lightmyography and electromyography in gesture glassification

In order to perform a comprehensive comparison of Electromyography and Lightmyography, the proposed LMG armband and a commercial EMG bioamplifier were used simultaneously for data collection during the execution of specific hand gestures. Participants performed the five gestures shown in Fig. 4 during data acquisition. We then used three machine learning techniques to train different classifiers and to predict the intended gesture of the user. The study was approved by the University of Auckland Human Participants Ethics Committee (UAHPEC), reference number #019043. All experiments were performed in accordance with relevant guidelines and regulations. Prior to the study, participants provided written and informed consent to the experimental procedures.

**Figure 5:**
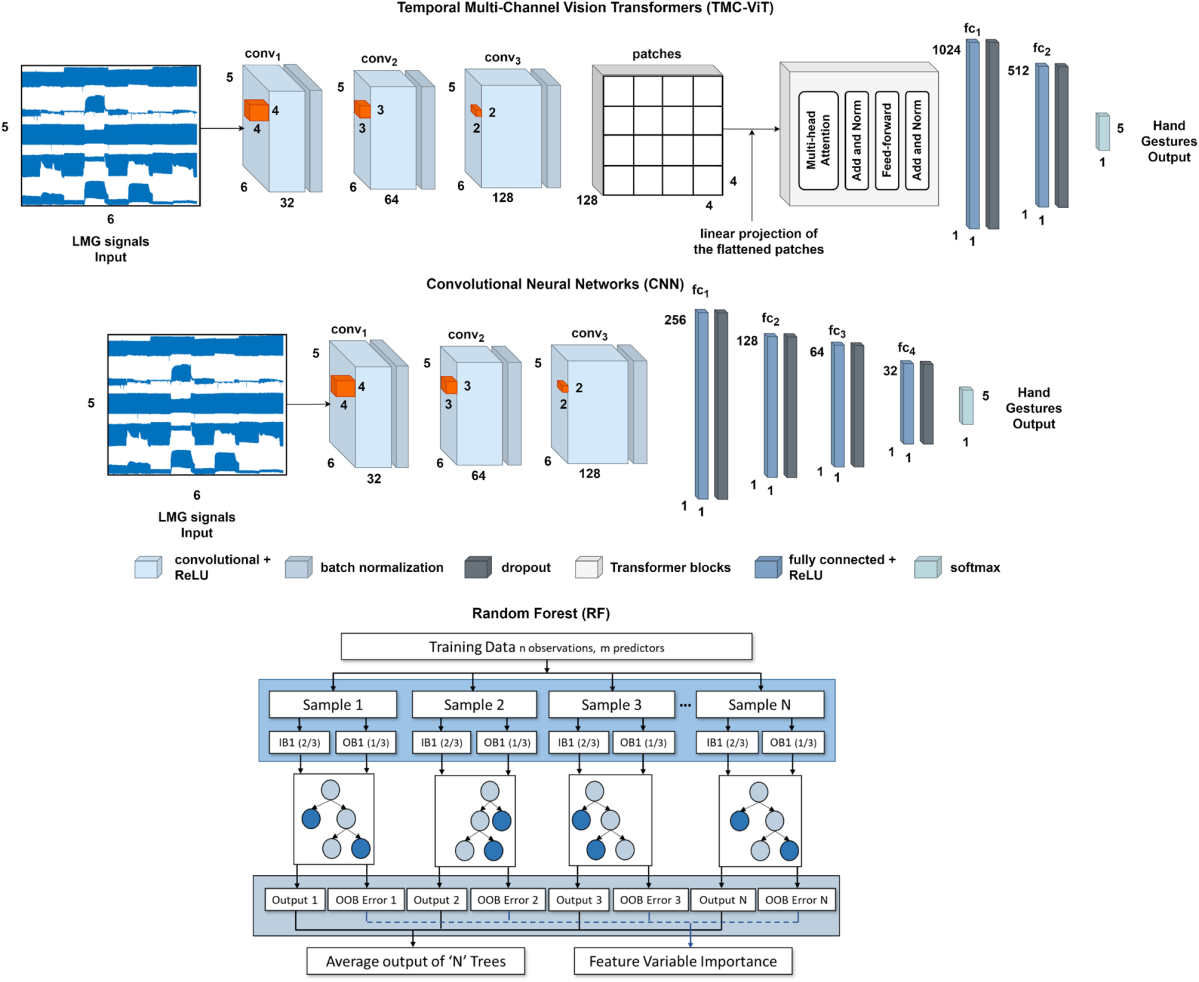
Temporal Multi-Channel Vision Transformers, Convolutional Neural Network, and Random Forest models developed to decode human hand gestures.

#### Data collection

For the gesture classification and recognition experiments, ten subjects participated in the data collection process. During data collection, the participants were asked to perform five different gestures: pinch, tripod, power, finger extension, and rest. These gestures were selected based on the frequent grasps identified by Bullock et. al.^35^. Figure 4 depicts these five gestures and provides an example of LMG and EMG recordings acquired during the execution of the gestures. Each session of data acquisition started with 15 seconds of rest followed by 15 seconds of gesture execution. This was repeated 6 times for each gesture. All the participants employed their dominant hand to execute the gestures. To develop machine learning models using supervised learning schemes, a software trigger was sent to the data recording script to label the gesture and rest phases. Similar to^36^, the LMG armband was worn on the upper half of the forearm, where the majority of the muscle groups involved in digit movement (extensor digitorum, flexor digitorum superficialis, flexor digitorum profundus, and flexor pollicis longus) are located^37^. In order to compare the performance of the LMG with state-of-the-art EMG, measurements from five bipolar EMG channels placed beside each LMG module were acquired at the same time as data from the armband using g.tec’s g.USBamp bioamplifier.

#### Data preprocessing

In this section, we present the data preprocessing steps required for the LMG and EMG-based gesture classification.

- *Sliding Window*: We employed a sliding window of 200 ms with a stride of 20 ms to extract samples from the LMG and EMG data collected. The sliding window was chosen to be larger than 125 ms to avoid high biases and variance^38^ and smaller than 300 ms due to real-time constraints of prosthetic control systems^39^.
- *Data Balancing*: The data was balanced to guarantee that we have the same number of samples for each of the five gestures so as to avoid bias toward a particular class.
- *Data Type*: The models were trained using raw LMG data without feature engineering. In contrast to EMG, the LMG data does not need to be filtered during acquisition. We also rely on the ability of the deep learning methods to automatically learn discriminative features from raw data, even with noisy signals. The EMG data was filtered using a 5 Hz and 500 Hz Butterworth bandpass filter. Eight time domain features were extracted from the raw EMG signals, namely: root mean square value, waveform length, zero crossings, mean absolute value, integrated EMG, Willison amplitude, variance of the EMG signal, and the log detector value. Details regarding the features can be found in^40^. The use of batch normalization layers within the deep learning models incorporates normalization into the architecture, increasing training speed and eliminating the need for normalization during preprocessing steps^41^.

#### Classification methods and models

In order to compare the performance of the LMG armband with the EMG bioamplifier, we trained appropriate models (shown in Fig. 5), employing three different machine learning techniques:

- *Random Forest (RF)*: RF is an ensemble classification method based on a combination of multiple decision trees. In this classic ML technique, the output is the most popular class among the decisions of individual trees^42,43^. RF models offer good predictive performance and are fast to train, at the cost of not being as robust as most deep learning techniques. In this paper, the RF model was used with 150 trees.
- *Convolutional Neural Network (CNN)*: CNNs are employed in several applications due to their ability to extract spatial characteristics and identify patterns of a given input data. CNNs represent the state-in-the-art in tasks ranging from classification to regression^44–47^. The CNN used in this paper comprises three convolutional blocks, four fully-connected layers, and a final softmax layer to predict the hand gestures. Each convolutional block is composed of convolutional, batch normalization, and dropout^48^ layers.
- *Temporal Multi-Channel Vision Transformer (TMC-ViT)*: This is a novel deep learning technique. The TMC-ViT^49^ is a Transformer-based model that adapts the Vision Transformer^50^ to process temporal data with multiple channels, e.g. LMG signals as input, employing convolutional and max-pooling layers to reduce the input dimension and extract its embeddings. Two convolutional layers are used before the data is supplied to a ViT that extracts $2\times 2$ patches and provides the output to a Transformer encoder composed of four Multi-head Attention layers^51^ with four heads each. The ViT processes the multi-channel signals with a linear projection of the flattened patches, whose components indicate low-dimensional correlations in the patches, and the Multi-Head Attention mechanism aggregates signal information across all layers.

These three machine learning techniques were chosen based on their results in our previous classification and regression works involving EMG and LMG signals^49,52–54^, as well as those found in the literature^45,55^. While RF and CNN are well-established machine learning techniques that can perform well, the TMC-ViT has been achieving outstanding results. The results obtained by these models will be compared in this work.

#### Training and evaluation

Our models were developed in Python using Tensorflow and Keras. The experiments were performed on the New Zealand eScience Infrastructure (NeSI) high-performance computing facilities that are equipped with appropriate GPUs. The classifiers were trained and validated using the 5-fold cross-validation method. During the training of each deep learning model, the loss function was the sparse categorical cross-entropy. Optimization was done using Adam^56^, with the efficiency of the trained model being assessed using accuracy. One model was trained for each dataset and subject examined.

**Figure 6:**
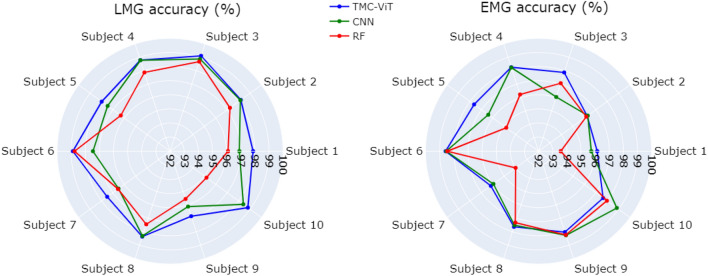
Decoding accuracy, in percentage, achieved by the TMC-ViT, CNN, and RF models. The accuracy achieved by the LMG-based models is shown on the left, and the EMG-based models are shown on the right.

### Lightmyography based force estimation

To further evaluate the potential applications of the proposed LMG armband, we focus on LMG-based grasping force estimation using the muscle contractions of the human upper forearm and collecting data from ten participants. The subjects selected to perform the force estimation experiment are not exactly the same as the ones that participated in the gesture classification experiment as separate sessions were held to avoid long duration of trials and therefore avoid muscle fatigue. This dataset was employed to train three regression models using raw LMG signals as input.

#### Data collection

To avoid experiencing fatigue while collecting adequate data, participants were asked to perform a single maximum force clench. They were then instructed to ramp up their clench force to half of the recorded maximum and reduce it back to rest during a 15-second period, following a 15 seconds period of resting. The 15-second periods were repeated 10 times and the force readings were simultaneously recorded with the LMG readings.

#### Data preprocessing

In this section, we present the data preprocessing steps required for the LMG-based force estimation.

- *Sliding Window*: Once again, for the reasons mentioned in the previous subsection, a sliding window of 200 ms with a stride of 20 ms was employed.
- *Data Balancing*: Only periods in which force was applied to the sensor were used to train and test the models.

#### Machine learning regression models

Three ML models were trained to perform force prediction: an RF regression model, a CNN, and the TMC-ViT. The model structure is the same as in Section 3.1.3, except for the last layer of the deep learning models. In order to perform regression, a dense layer with one neuron and linear activation function were employed.

#### Training and evaluation

The regression models were trained and validated using 10-fold cross-validation using one separated repetition for testing per fold. The mean squared error (MSE) loss function was employed during the training of each deep learning model. The efficiency of the trained regression models is assessed using the Pearson correlation coefficient and the percentage of the normalized mean square error (NMSE) representing accuracy in comparing the predicted and the actual force. The NMSE value of 0% denotes a bad fit, whereas the NMSE value of 100% denotes that the two trajectories are identical. The NMSE value is derived as follows

3 $$\begin{aligned} NMSE(\%) = 100*\left( 1 - \frac{\left\Vert x_{r}-x_{p}\right\Vert ^{2}}{\left\Vert x_{r}-mean(x_{r})\right\Vert ^{2}}\right) \end{aligned}$$

where $\left\Vert .\right\Vert$ indicates the 2-norm of a vector, $x_{r}$ is the actual reference motion, and $x_{p}$ refers to the predicted force.

## Results and discussion

In this section, we discuss the findings from the experiments conducted with the proposed LMG armband. More precisely, we compare the gesture decoding accuracies of the LMG armband with the accuracies provided by the models trained with the EMG data collected using a commercially available EMG bioamplifier. Furthermore, we also evaluate the performance of the LMG armband in decoding continuous grasping forces by measuring the contractions of the muscles of the human upper forearm.

**Table 2:** Gesture decoding and force estimation results.

HMI	LMG			EMG			Subj. Info		
Model	RF	CNN	TMC-ViT	RF	CNN	TMC-ViT	Sex	Age	Hand
Accuracy, in percentage, for the three gesture decoding models using LMG and EMG data for each subject (Subj.). The HMI with the higher accuracy is highlighted in bold.									
Subj. 1	$\varvec{96.10\pm 3.35}$	$\varvec{96.91\pm 2.63}$	$\varvec{97.88\pm 1.79}$	93.57±2.39	95.73±1.81	96.15±2.01	M	33	R
Subj. 2	$\varvec{97.24\pm 0.74}$	$\varvec{98.15\pm 0.53}$	$\varvec{98.19\pm 0.51}$	96.17±1.70	96.30±2.54	96.25±1.09	M	23	R
Subj. 3	$\varvec{98.68\pm 0.49}$	$\varvec{98.88\pm 0.37}$	$\varvec{99.11\pm 0.39}$	97.07±1.16	96.04±1.21	97.87±0.38	F	22	R
Subj. 4	$\varvec{97.87\pm 2.62}$	$\varvec{98.78\pm 0.42}$	$\varvec{98.79\pm 0.61}$	96.23±0.70	98.24±0.70	98.26±0.80	M	22	R
Subj. 5	$\varvec{96.31\pm 1.83}$	$\varvec{97.46\pm 1.70}$	$\varvec{97.98\pm 0.71}$	94.83±2.52	96.40±1.63	97.64±0.27	M	26	R
Subj. 6	$\varvec{98.76\pm 0.34}$	$\varvec{98.81\pm 0.18}$	$\varvec{98.88\pm 0.31}$	98.46±0.79	98.53±0.58	98.58±0.49	M	25	R
Subj. 7	$\varvec{96.56\pm 0.56}$	$\varvec{96.51\pm 0.87}$	$\varvec{97.50\pm 0.37}$	94.01±3.00	95.95±1.62	96.18±1.14	M	31	R
Subj. 8	$\varvec{97.45\pm 1.12}$	$\varvec{98.32\pm 0.55}$	$\varvec{98.37\pm 0.58}$	97.32±2.01	97.52±0.71	97.64±0.49	M	30	R
Subj. 9	95.56±1.29	96.13±0.79	96.85±0.49	$\varvec{98.24\pm 0.85}$	$\varvec{98.27\pm 0.56}$	$\varvec{98.03\pm 0.54}$	M	23	R
Subj. 10	95.19±4.76	98.41±0.37	$\varvec{98.81\pm 0.28}$	$\varvec{97.98\pm 1.42}$	$\varvec{98.85\pm 0.86}$	97.65±1.96	F	23	L
AVG	${\underline{{{\textbf {96.97}}}\pm {{\textbf {1.71}}}}}$	${\underline{{{\textbf {97.84}}}\pm {{\textbf {0.84}}}}}$	${\underline{{{\textbf {98.24}}}\pm {{\textbf {0.60}}}}}$	${{96.39}}\pm {{1.65}}$	${{97.18}}\pm {{1.23}}$	${{97.42}}\pm {{0.92}}$			

Model	RF		CNN		TMC-ViT		Subj. Info		
Metric	C	A	C	A	C	A	Sex	Age	Hand
Correlation (C) and accuracy (A), in percentage, for the three force regression models using LMG for each subject.									
Subj. 1	89.82±3.45	78.14±5.52	95.52± 2.36	89.79±4.22	96.16±3.66	92.32±6.72	M	33	R
Subj. 2	90.76±4.09	75.65±8.22	96.81± 0.80	90.56±4.57	98.07±0.72	94.26±2.26	M	23	R
Subj. 3	83.27±5.39	56.87±25.79	94.18±4.60	88.03±8.00	95.52±3.99	88.08±8.51	F	22	R
Subj. 4	94.17±7.03	88.51±12.94	98.77±0.74	87.14±1.57	99.15±0.33	98.09±0.72	M	22	R
Subj. 5	82.89±11.7	71.71±16.12	89.68±4.39	78.40±6.47	92.22±4.14	83.34±8.10	M	28	L
Subj. 6	87.36±4.44	71.07±5.53	94.29±3.79	88.48±7.39	95.36±1.61	90.65±2.95	M	25	R
Subj. 7	94.51±4.11	87.02±3.72	96.90±1.21	91.40±3.05	96.96±0.74	92.43±3.33	M	25	L
Subj. 8	96.25±2.85	90.37±7.61	97.22±2.45	94.30±4.21	98.46±0.37	96.79±0.45	M	30	R
Subj. 9	79.45±8.40	62.96±12.58	83.01±6.51	72.19±7.88	89.88±4.59	74.06±5.89	M	26	R
Subj. 10	96.15±2.00	84.82±14.65	97.14±1.02	93.52±2.09	97.48±1.01	94.56±2.39	F	23	L
AVG	${{\textbf {89.47}}}\pm {{\textbf {5.71}}}$	${\textbf {76.71}}\pm {\textbf {10.68}}$	${{\textbf {94.35}}}\pm {{\textbf {4.47}}}$	${\textbf {88.38}}\pm {\textbf {7.18}}$	${{\textbf {95.93}}}\pm {{\textbf {2.75}}}$	${\textbf {90.46}}\pm {\textbf {6.80}}$			

### LMG and EMG gesture classification comparison

The gesture classification accuracies of the models trained with the data collected with the LMG armband and the EMG bioamplifier for the three ML classifiers examined are all presented in Table 2. It can be seen that all the tested ML techniques presented better gesture decoding accuracies when LMG data was used as input, except for the models trained using subject 9 data and two models using subject 10 data. To validate the significance results, an analysis of variance (ANOVA) was performed, comparing the accuracies of the models developed using LMG data and EMG data. The null hypothesis of ANOVA was that there is no difference between the results achieved by the LMG and EMG-based models. A *p-*value of 0.0381 was achieved, rejecting the null hypothesis and therefore showing that the results are statistically significant (*p-*value< 0.05). Moreover, the CNN and TMC-ViT LMG-based models show a smaller standard deviation than those trained using feature extracted EMG, indicating more consistent and robust results among the tested subjects. It is worth noting that raw LMG signals are directly supplied to the models, unlike the myoelectric activations, which need to go through feature engineering steps. Eliminating additional feature extraction steps that require a certain amount of computational power is of paramount importance, especially in real-time applications where minimizing sample processing time is critical. Moreover, considering the bioamplifier size and weight, feature extracted EMG is not a suitable solution for portable applications, while the LMG armband provides an ideal solution for such applications due to its simple components, small size, lightweight, and low cost.

The female participants achieved an average accuracy of 98.98% for the TMC-ViT model using LMG signals as input, against 98.05% achieved by the male participants. When analyzing the handedness, left-handed participants achieved an accuracy of 98.24% against 98.17% for right-handed participants. Based on the results, variables such as handedness and gender of the participant do not seem to interfere with the results obtained by the LMG armband. However, future work will systematically assess these variables with a more extensive and diverse group of participants.

As can be seen in Fig. 6, the TMC-ViT achieved the highest accuracy values for all tested subjects, followed by the CNN and the RF, respectively. The results achieved by the models for LMG signals are higher in terms of accuracy and more consistent in terms of standard deviation when compared to EMG. The TMC-ViT achieves a classification accuracy of up to 99.11% for raw LMG data (see Table 2).

### LMG based force estimation

**Figure 7:**
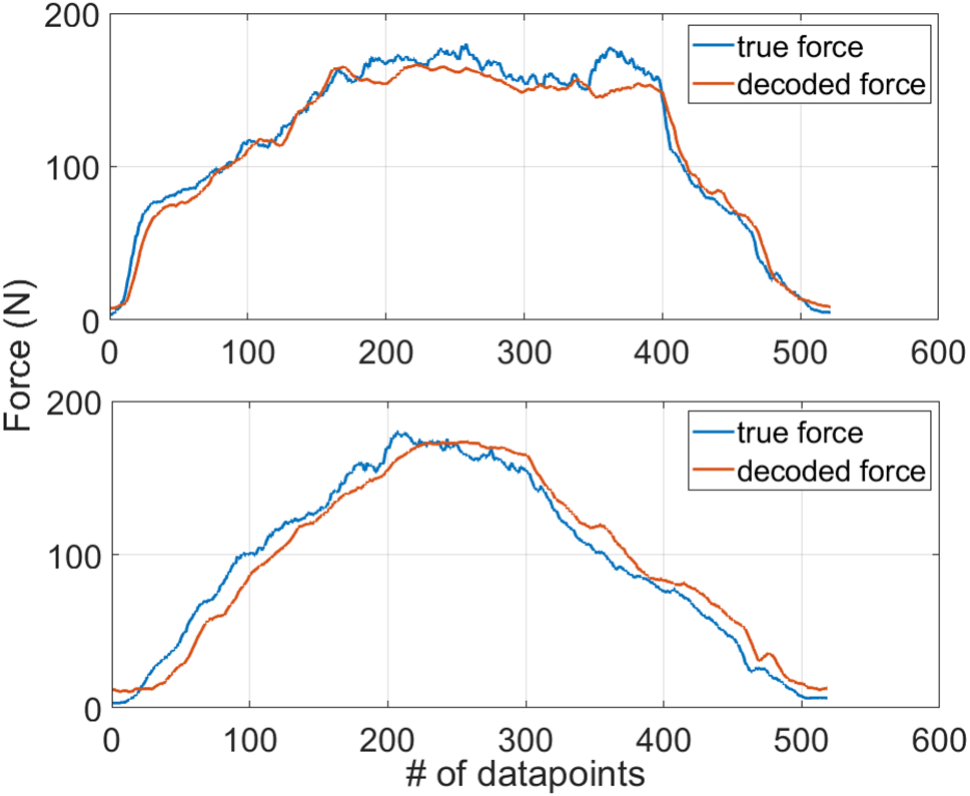
Two examples of decoded clenching force vs the actual clenching force using the LMG data. The forces were decoded using the TMC-ViT model. True force is shown in blue and decoded force is shown in red.

The correlation and accuracy achieved by the RF, CNN, and TMC-ViT models for predicting the clenching force are shown in Table 2. The TMC-ViT achieved the highest correlation and accuracy and lowest standard deviation between the tested machine learning techniques, followed by the CNN and the RF, as expected for being a deeper and more complex model. The RF, a classic machine learning technique, presented the worst performance among the tested machine learning methods. Deep learning methods could achieve better correlation and accuracy, demonstrating their robustness with the availability of large datasets. The regression results validate that the clenching force can be decoded employing only raw LMG data as input. With the proposed LMG armband, the force decoding accuracies achieved were as high as 98.09% while the correlation of the decoded and actual forces was as high as 99.15%, demonstrating the outstanding performance of the LMG armband in decoding clenching forces. In Fig. 7, we show the decoded and true clenching force of two different trials as decoded by the TMC-ViT model.

### LMG armband design considerations

Based on the results of the experiments presented in Section 2.1, it can be concluded that the main factors that affect the signal quality of each module are wavelength of the LEDs and the distance of the LED from the skin. Shorter wavelengths penetrate the skin less, resulting in better reflection, leading to a more sensitive response. On the other hand, longer wavelengths can penetrate the skin deeper and provide complementary data that can be beneficial for decoding the user’s intention. With longer wavelengths, it is also possible to acquire some health information, which can be a potential advantage of light-based myography over classic EMG techniques^57^. The distance of the LED from the skin also changes the signal response noticeably. Shorter wavelengths require a minimum distance from the skin to maximize the reflection area, to effectively capture the skin displacements. However, with a longer wavelength, it is better for the source to be as close as possible to the skin or in contact with the skin to achieve better penetration. As colored silicone may absorb or reflect light, a transparent silicone medium is more suitable between the light sources and the skin, however environmental lighting should be blocked out. Furthermore, flexible reflective surfaces can improve the signal when short wavelengths (e.g., when using green light) are used, but they cannot be used with longer wavelengths (e.g., when using IR), since they don’t allow the light to penetrate the skin.

## Conclusion

In this work, we introduced a new muscle-machine interfacing method called Lightmyography (LMG). LMG employs light that travels through elastic media and human tissue, and measures the changes in light luminosity so as to detect muscle movement and the deformations of the surrounding tissue that affect light absorption and reflection. Similar to another muscle-machine interface called Forcemyography, LMG detects muscle contraction through tissue deformation and skin displacements. In this study, we systematically explored the effects of the different light wavelengths and LMG module configurations on gesture classification and force decoding performance. In particular, the use of a silicone layer between the light source and the skin was considered and the effect of the silicone layer thickness, stiffness, and colour was studied. Based on our analysis, we derived the light wavelength, distance of the sensor from the skin, silicone layer thickness, and silicone stiffness that produce the best results. An LMG armband that consists of five sensing modules was developed to facilitate data collection. Each LMG module has one green LED, one IR LED, and one photodiode in conjunction with an operational amplifier unit. The efficiency of the proposed LMG armband has been experimentally validated, comparing the performance of machine learning models trained with the data collected using the LMG armband with that of models trained with data collected by a commercially available EMG bioamplifier. Three different machine learning techniques (RF, CNN, and TMC-ViT) were employed to develop the motion, intention, and force decoding models, achieving gesture classification accuracies of up to 98.24% (for the TMC-ViT model) and force decoding accuracies of up to 90.46% (again for the TMC-ViT model), using LMG data. Thus, it was shown that the LMG-based models could successfully discriminate between five different gestures that are commonly used in everyday life scenarios and can efficiently estimate the exerted grasping forces. It was also demonstrated that models developed using LMG data outperformed the models trained with EMG data for every machine learning technique examined. It should also be noted that the LMG armband is lighter, smaller, and cheaper to produce compared to commercially available EMG bioamplifiers, making it more attractive for wearable applications that require portability of the system.

In future studies, we plan to explore the applicability of LMG in health monitoring (e.g, detection of heartbeat, blood oxygen level, etc.). We also plan to integrate inertial measurement units (IMUs) in the armband so as to improve both gesture recognition and force decoding in various arm orientations, triggering configuration-specific models. Finally, we plan to further investigate the effect of fatigue on the motion, intention, and force decoding performance.

## Data Availability

The datasets generated during and/or analysed during the current study are available from the corresponding author on reasonable request.
